# Supplementary material for: Sodium enhances indium-gallium interdiffusion in copper indium gallium diselenide photovoltaic absorbers
Source: Nat Commun. 2018 Feb 26;9:826. doi: 10.1038/s41467-018-03115-0 (PMC5827571; doi:10.1038/s41467-018-03115-0)
Supplement: Supplementary file 1 — Supplementary Information [file 41467_2018_3115_MOESM1_ESM.pdf]

Supplementary figures

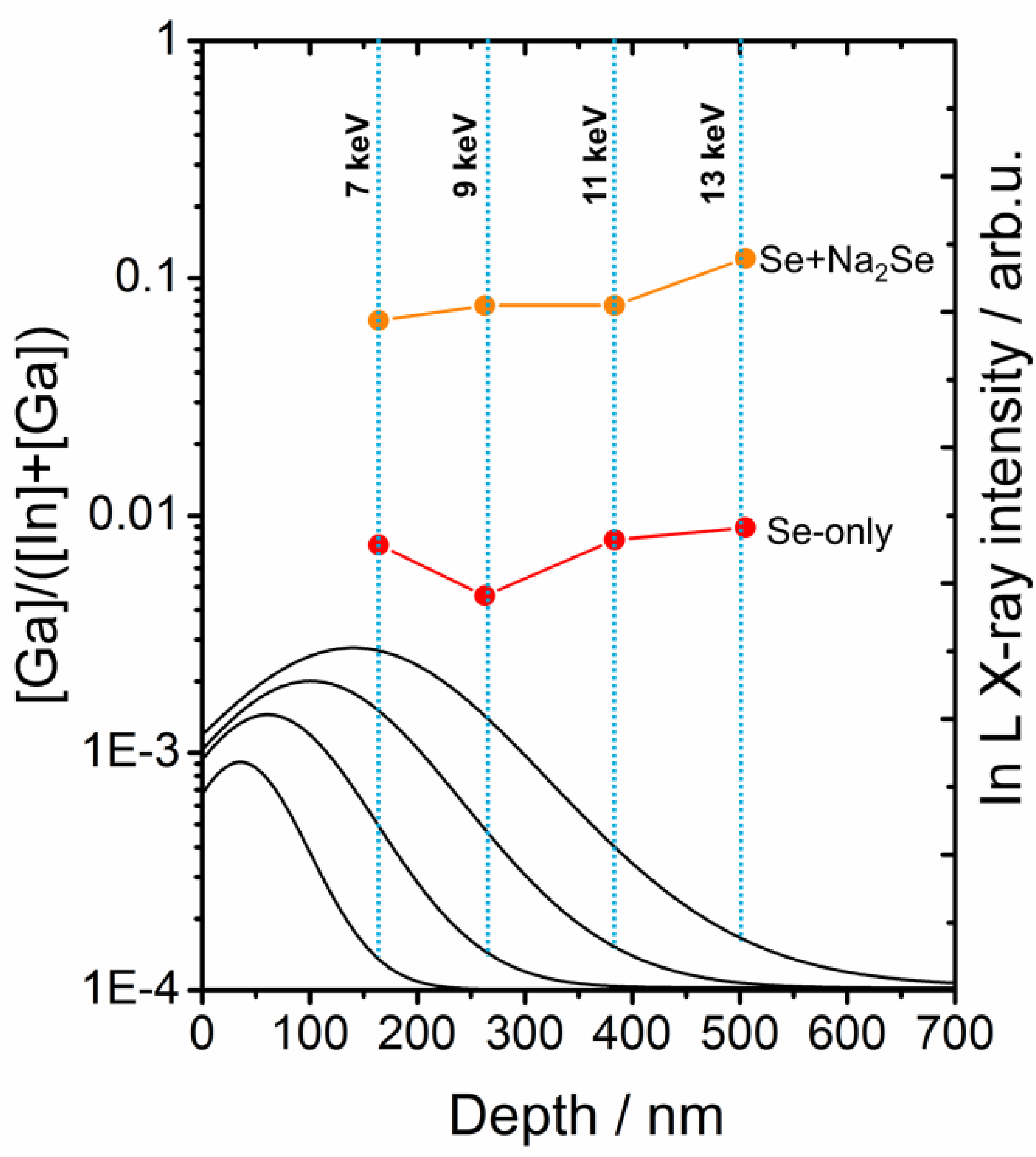

Supplementary Fig. 1| Reconstructed EDS depth profiles of CIS/GaAs samples. Ga/(In+Ga) atomic ratio of *Se-only* and *Se+Na$_2$Se* films obtained by SEM-EDS in the range 7-13 keV. The reconstructed depth information is plotted as the maximum + 2σ of the Gaussian fitting of the In L X-ray line emission distribution simulated with Casino ©[1].

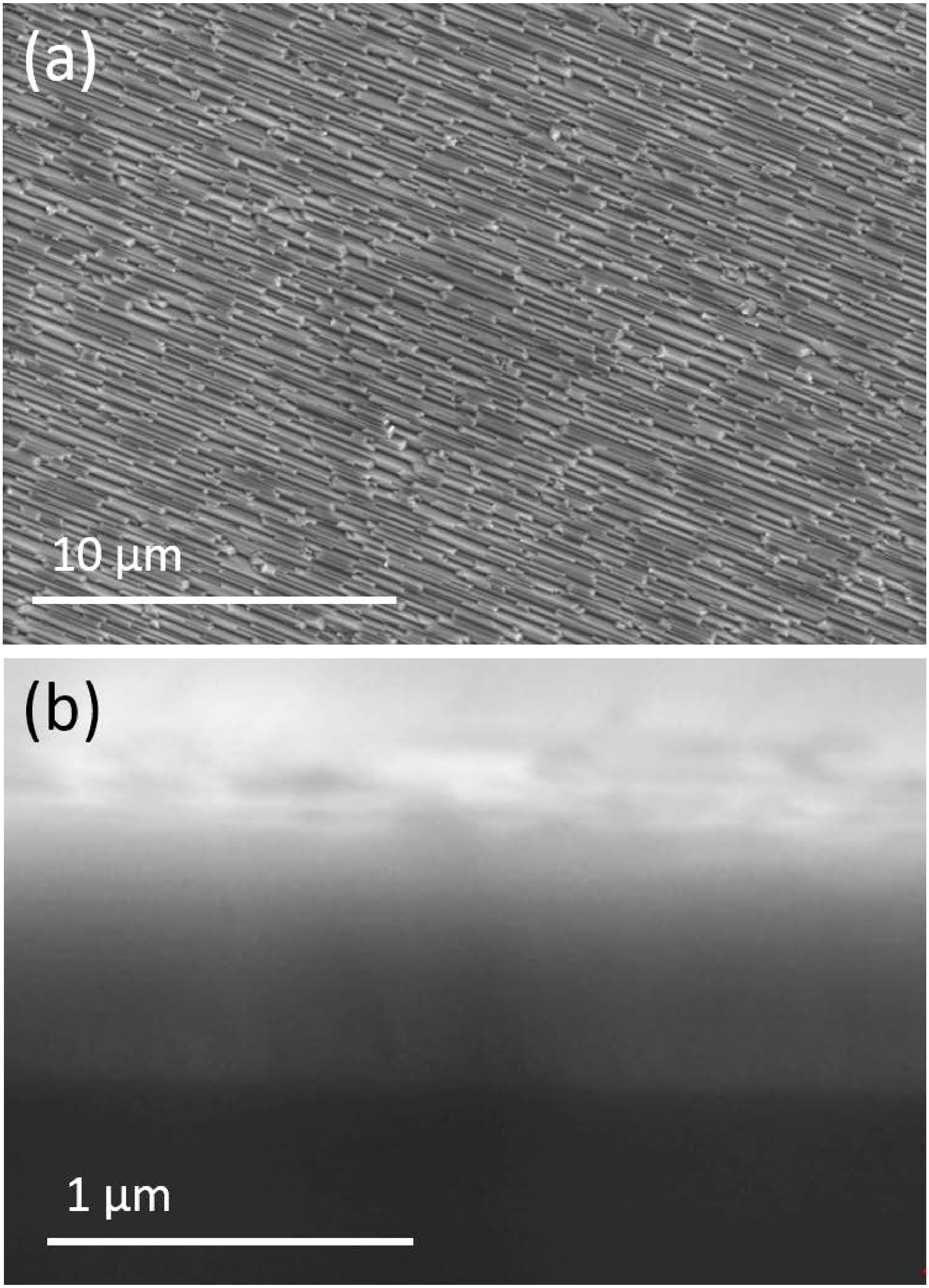

Supplementary Fig. 2| Top view image (a) and cross section (b) SEM images of the *Untreated* sample

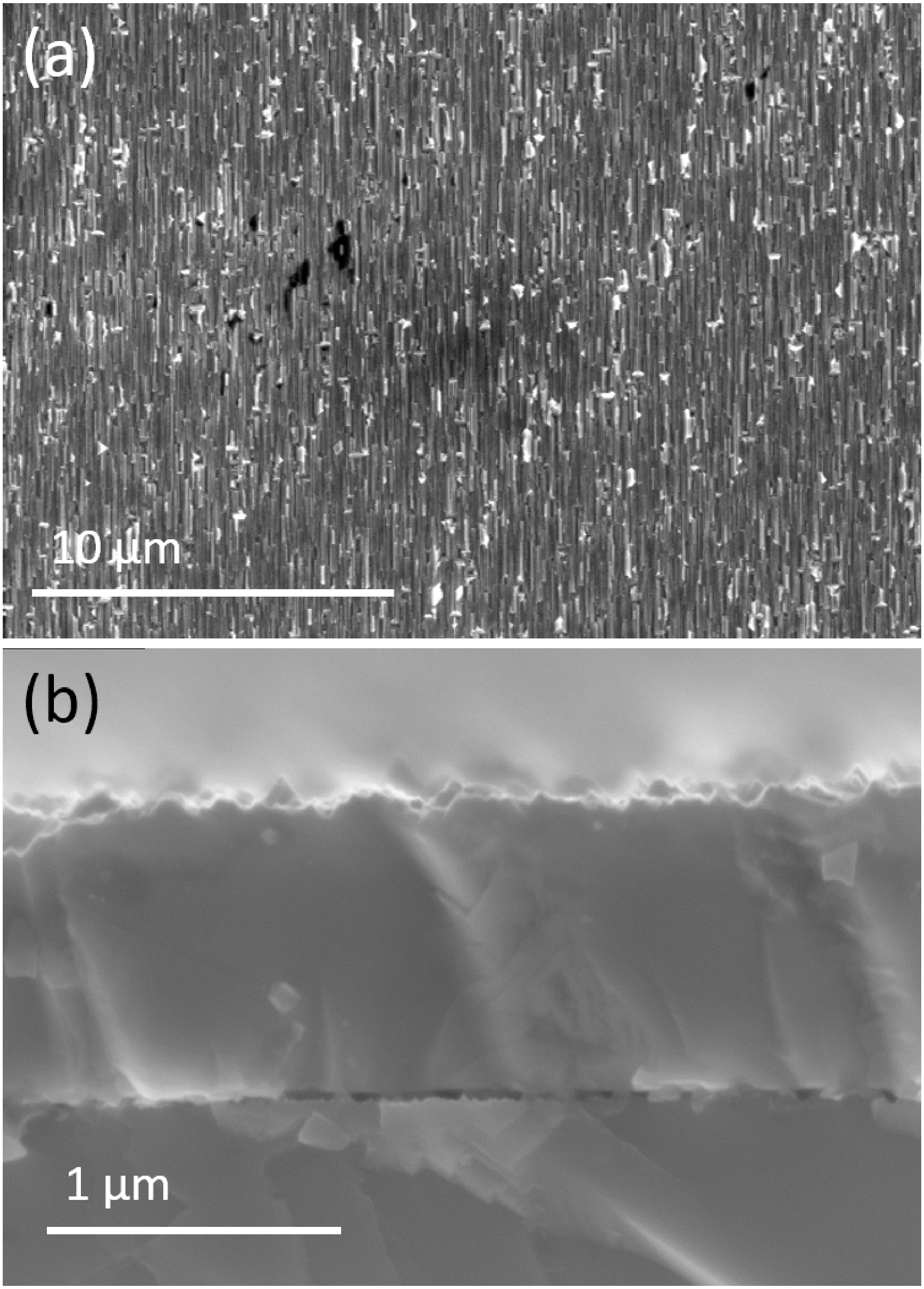

Supplementary Fig. 3| Top view image (a) and cross section (b) SEM images of the *Se-only* sample

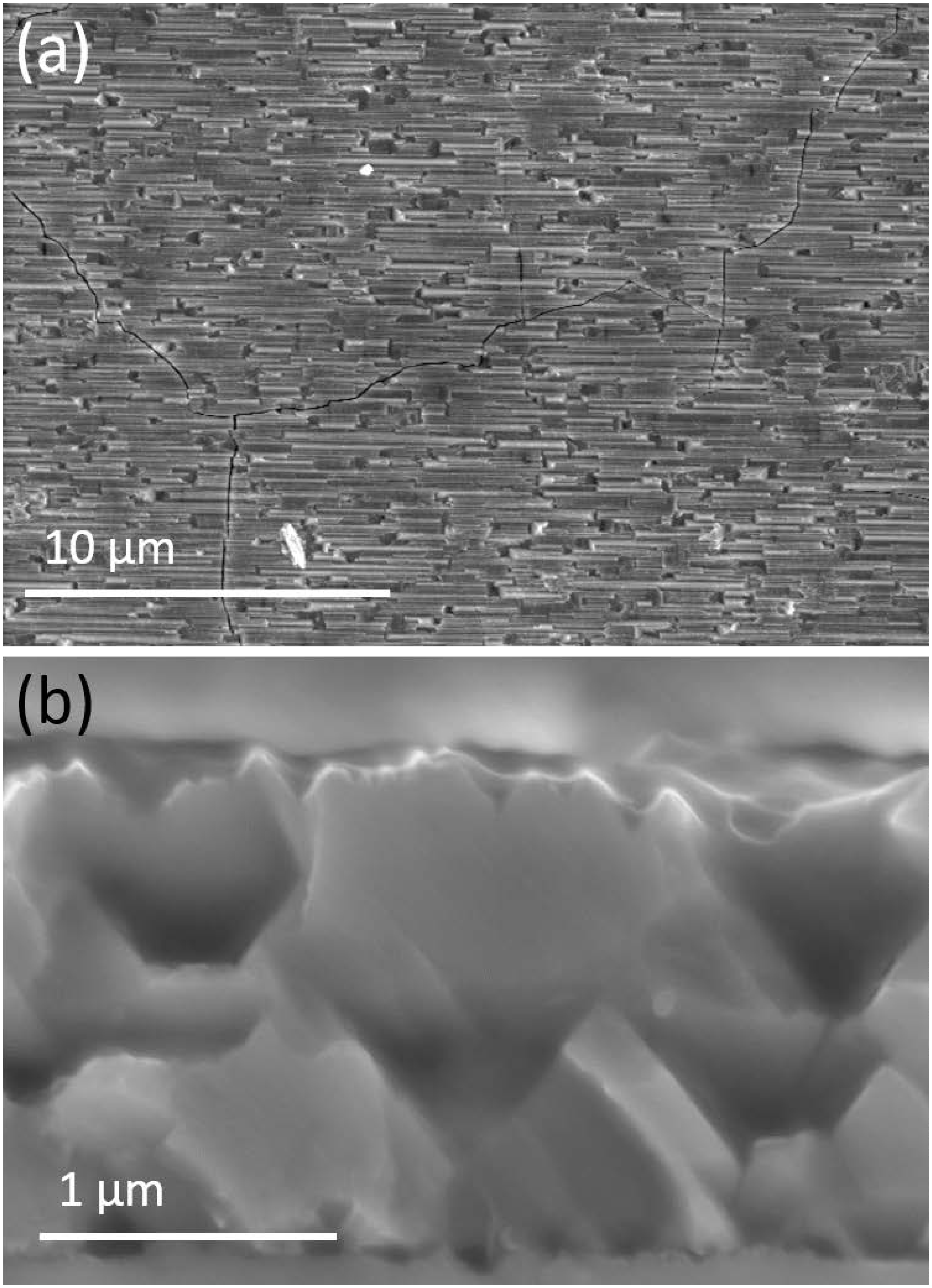

Supplementary Fig. 4| Top view image (a) and cross section (b) SEM images of the *Se+Na_2Se* sample

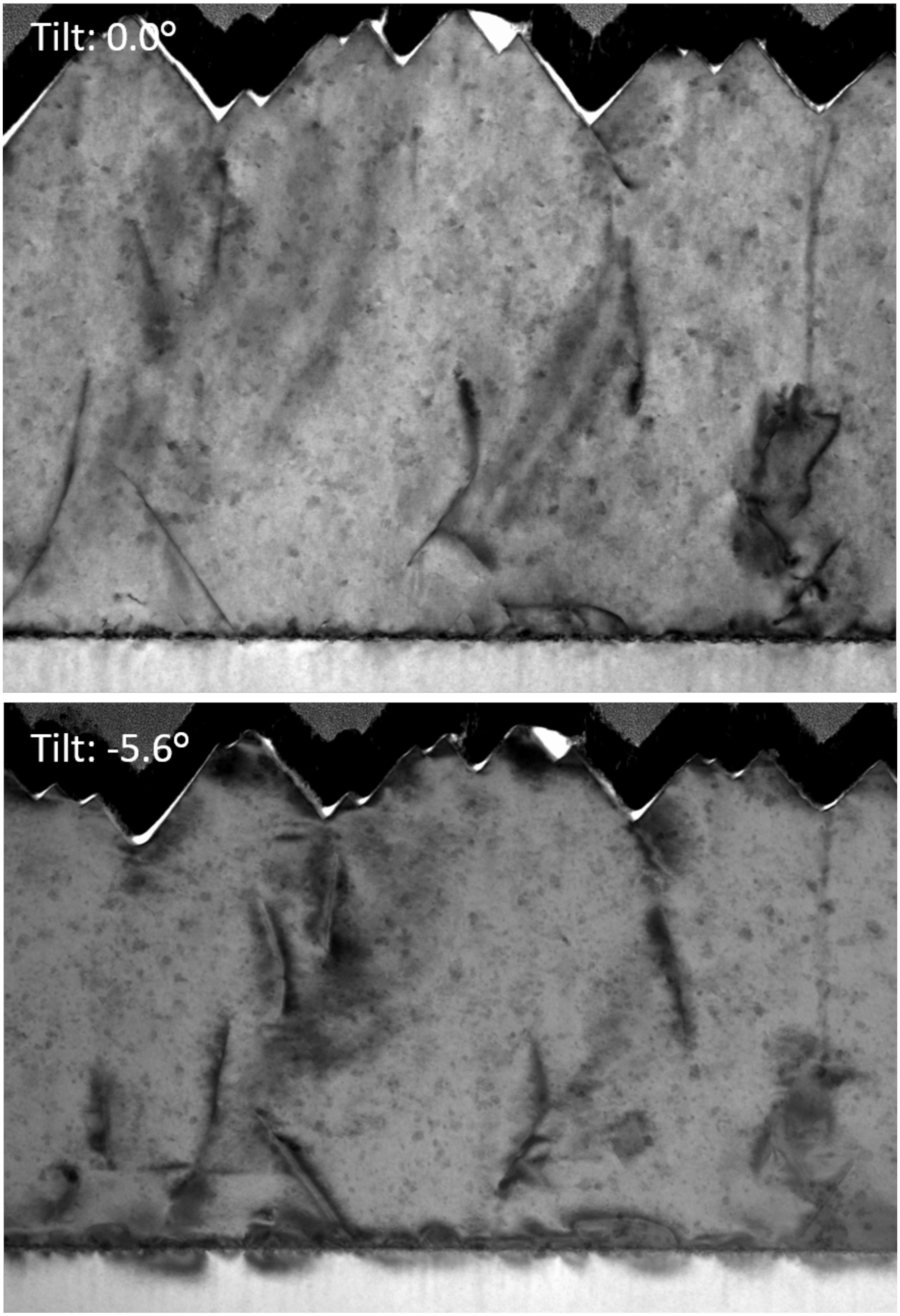

Supplementary Fig. 5| Cross section STEM images of the *untreated* sample at 0.0 and -5.6 deg. tilt.

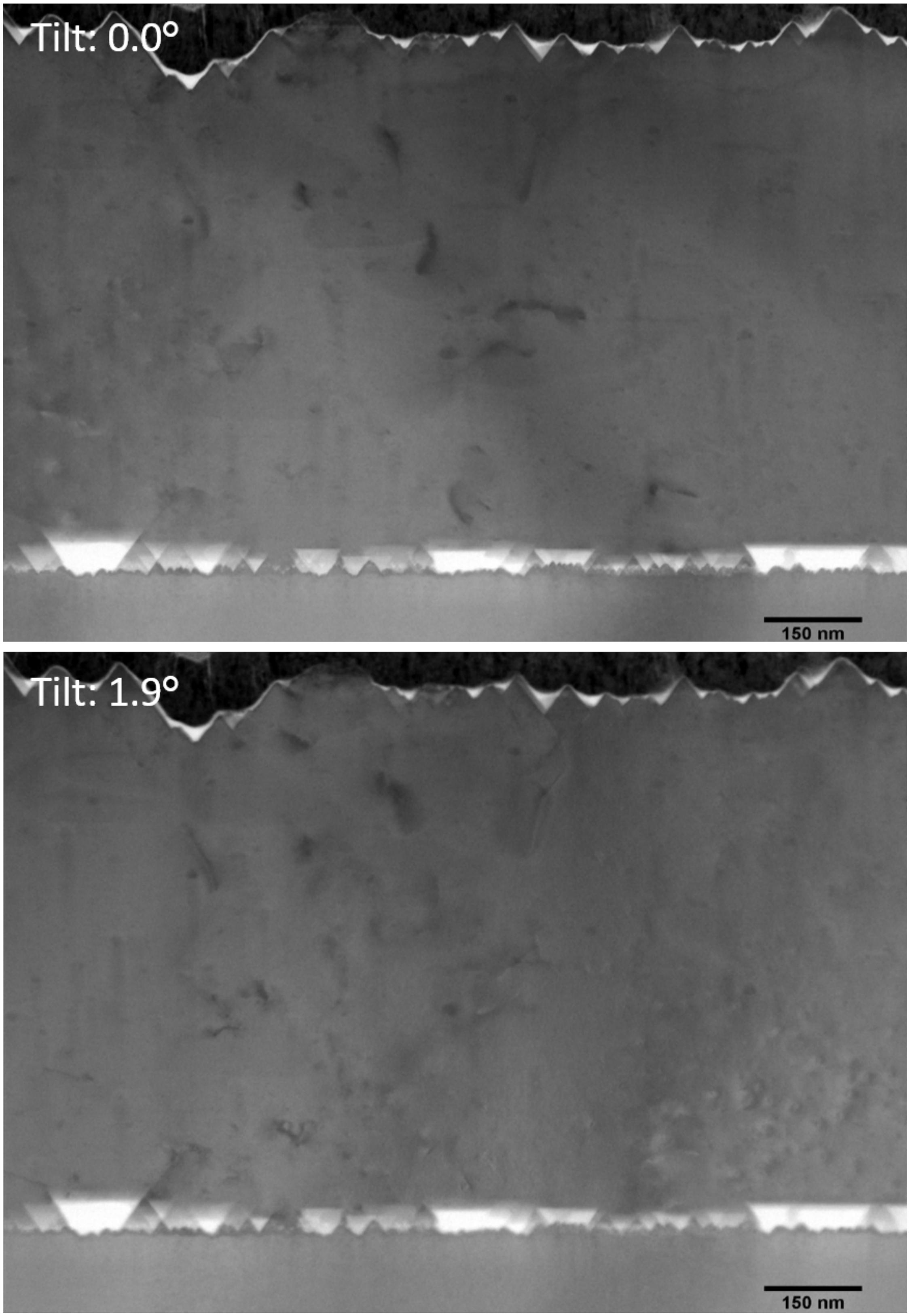

Supplementary Fig. 6| Cross section STEM images of the *Se-only* sample at 0.0 and 1.9 deg. tilt.

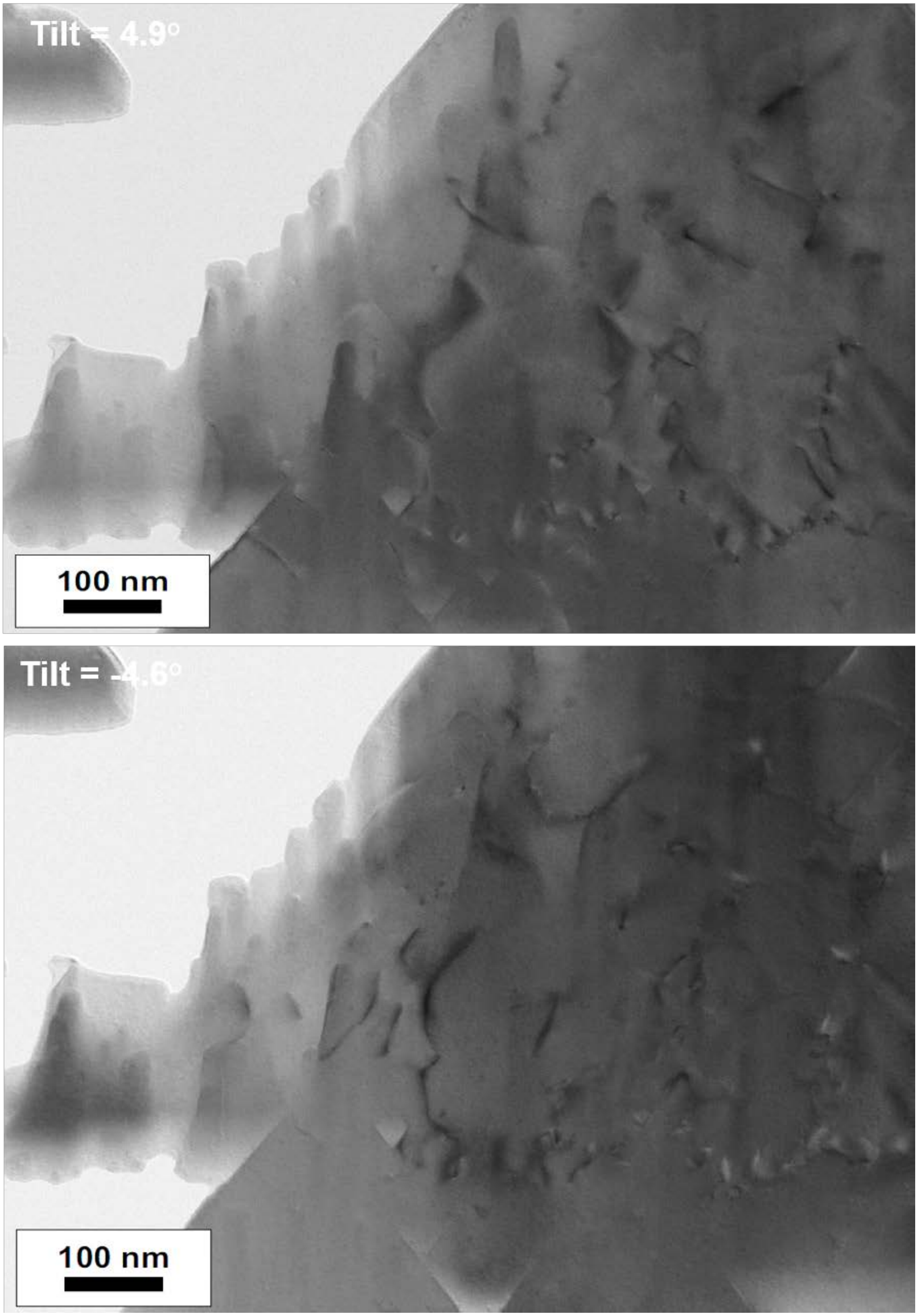

Supplementary Fig. 7| Cross section STEM images of the *Se-only* sample at -4.9 and -4.6 deg. tilt.

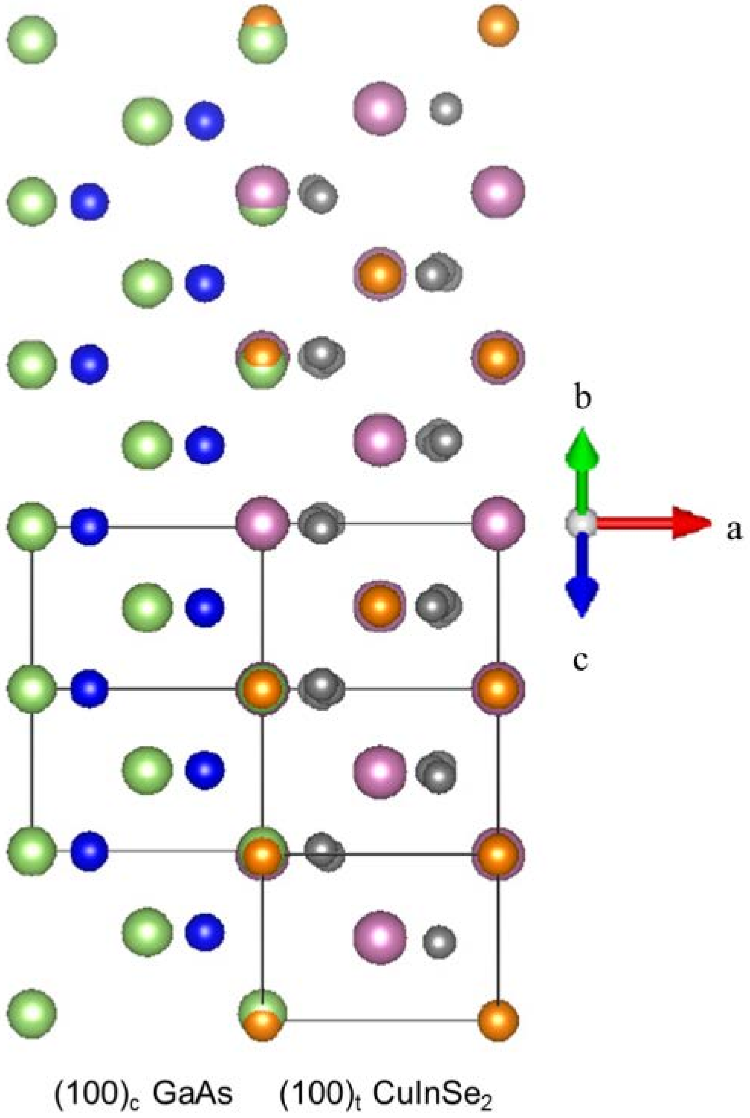

	Misfit strain *e*	Strained parameter
**a_{film}//a_{subs}**	$\frac{a_{GaAs} - a_{film}^{bulk}}{a_{film}^{bulk}}$	$c_{st} = \left(\frac{a_{bulk}}{a_{st}}\right)^2 \cdot c_{bulk}$
**c_{film}//a_{subs}**	$\frac{a_{GaAs} - c_{film}^{bulk}/2}{c_{film}^{bulk}/2}$	$a_{st} = \left(\frac{c_{bulk}}{c_{st}}\right)^{1/2} \cdot a_{bulk}$

Supplementary Fig. 8| $(100)_c$ GaAs / $(100)_t$ CIS misfit strain representation and calculation.

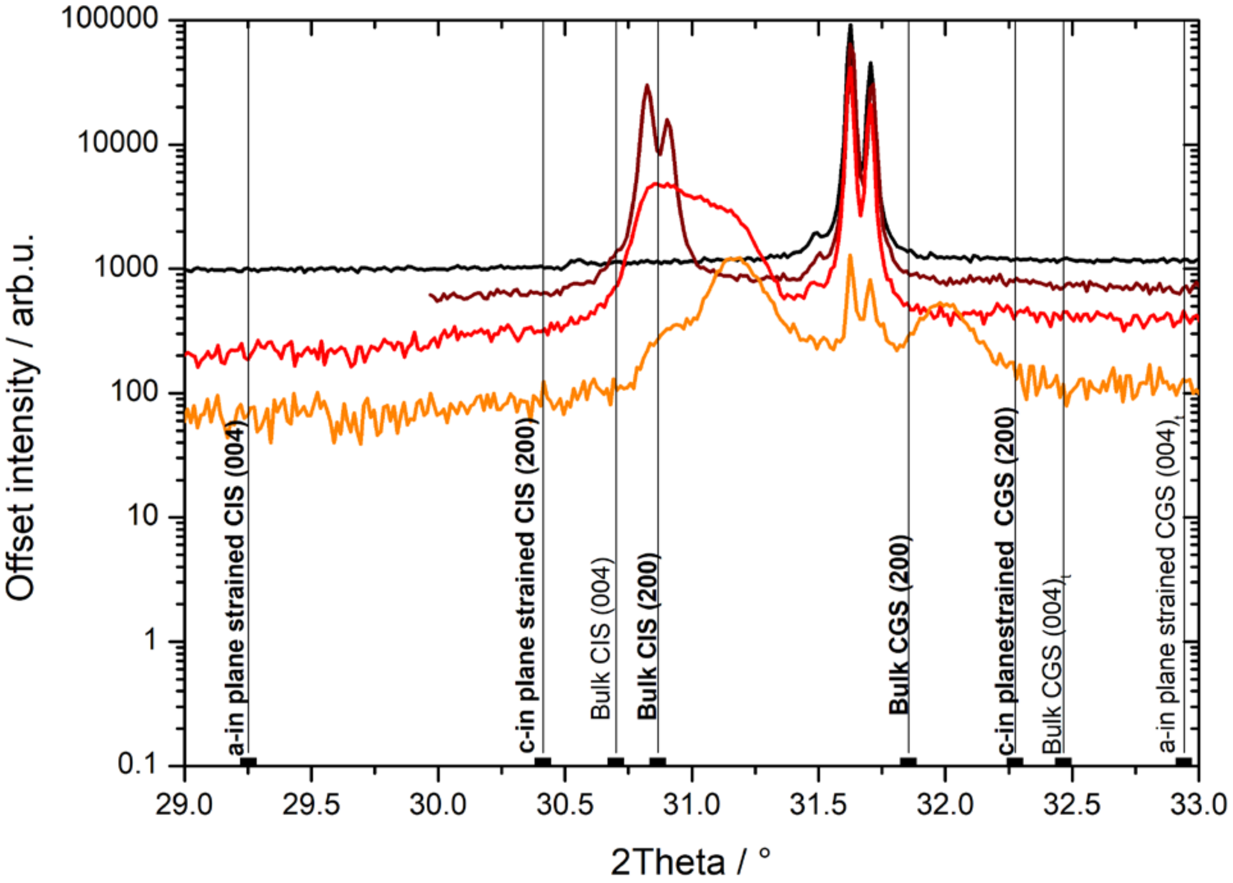

	**CIS**	**CIS** [2]	**CGS** [3]
***a* / Å**	5.784	5.789	5.614
***c* / Å**	11.621	11.639	11.022
**e_a / %**	-2.251	-2.335	+0.709
**e_c / %**	-2.697	-2.847	+2.591
**$\{200\}_{t, bulk}$ / 2θ**	30.89	30.87	31.85
**$\{200\}_{t, st}$ / Å**	5.864	5.873	5.543
**$\{200\}_{t, st}$ / 2θ**	30.46	30.41	32.23
**$\{004\}_{t, bulk}$ / 2θ**	30.75	30.70	32.47
**$\{004\}_{t, st}$ / Å**	12.162	12.202	10.867
**$\{004\}_{t, st}$ / 2θ**	29.35	29.25	32.94

Supplementary Fig. 9| Position of bulk and fully strained reflections of CIS and CGS grown on (100) GaAs, along with the experimental diffractograms. Calculated misfit strains and positions in the diffractograms of $(200)_t$ and $(004)_t$ reflections for the different crystallographic orientations of CIS and CGS on (100) GaAs (*a*=5.6538[4]), assuming λ(Cu $K_{\alpha 1}$) = 1.54056 Å.

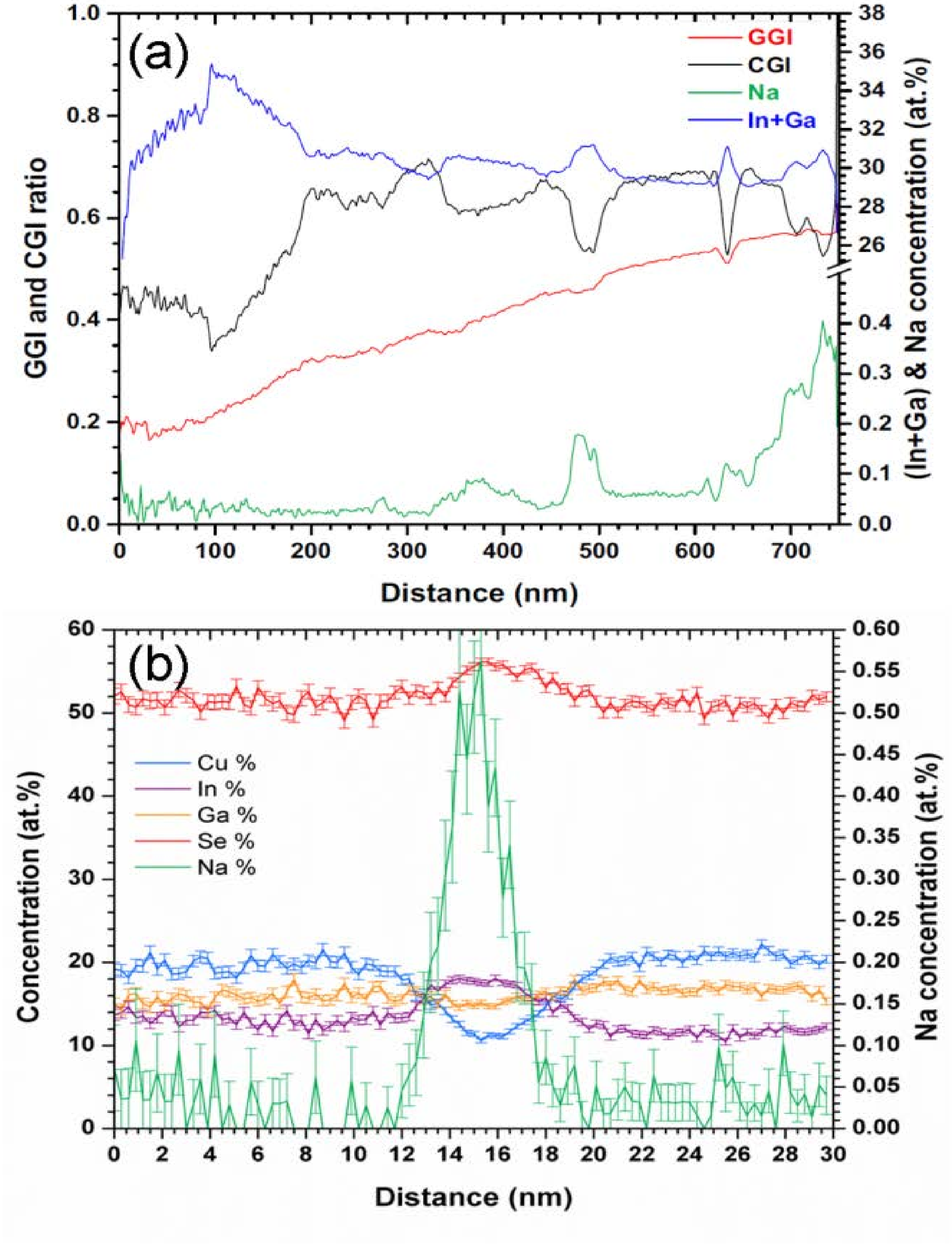

Supplementary Fig. 10| Reconstructed APT depth profiles. Depth profiles of Na, In+Ga, CGI and GGI averaged over the entire APT tip (a) and across a likely planar defect (b) of the *Se+Na$_2$Se* film.

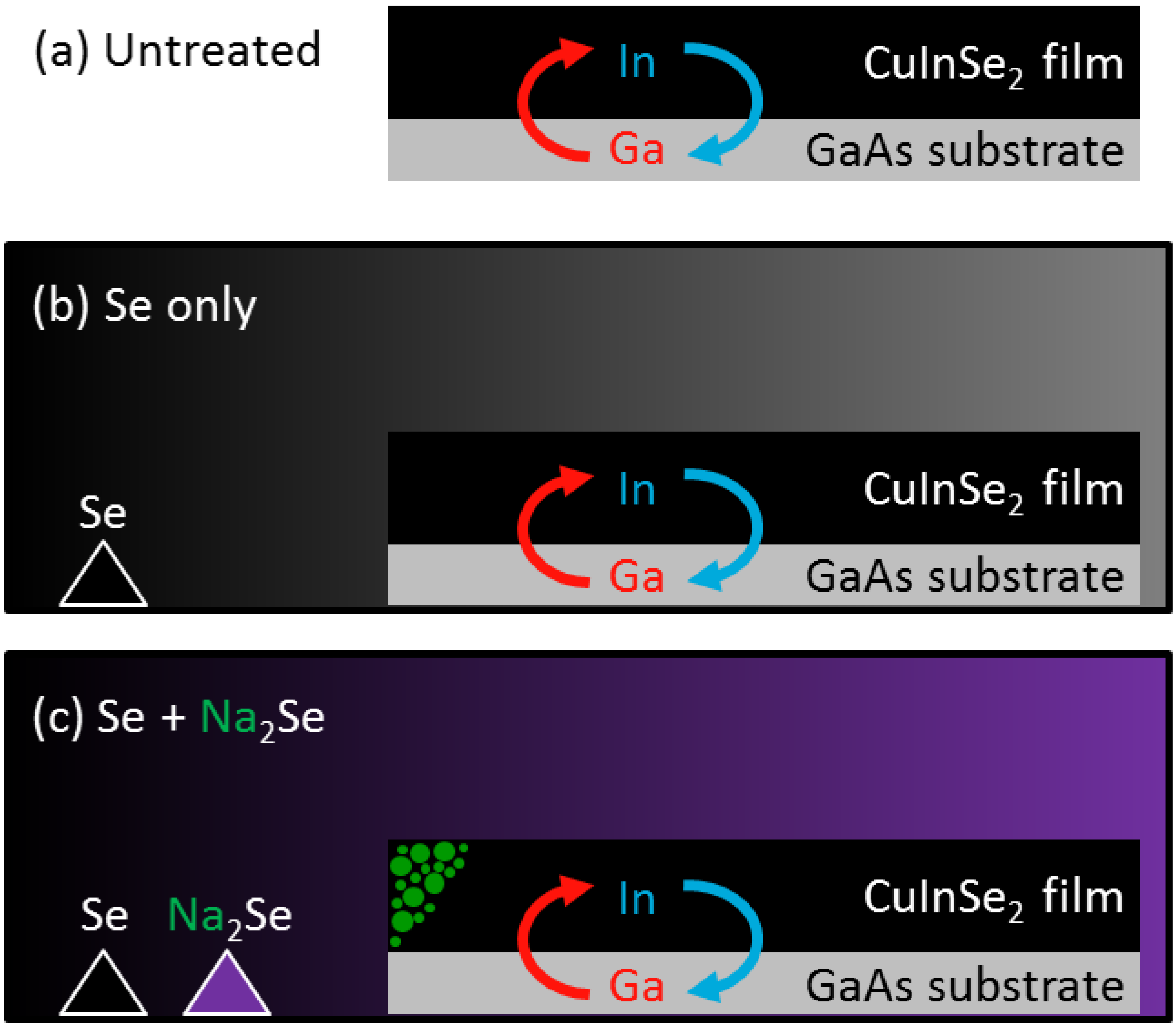

Supplementary Fig. 11 | Schematics of Na gas-phase doping experiment. Schematics of CIS/GaAs diffusion couple experiments. The extent of In-Ga interdiffusion is assessed on (a) a sample as obtained from the MOCVD at 470 °C (*untreated*) and on samples annealed at 570 °C for 30 minutes in the presence of (b) 100 mg of just selenium (*Se-only*) or (c) 100 mg of selenium and 10 mg of sodium selenide (*Se+*Na_2Se). The annealings are performed inside a graphite box placed in a tubular furnace kept at 10 mbar of 10 % H_2 in N_2.

Supplementary discussions

Supplementary discussion 1 | Theoretical misfit strains of epitaxial films.

Due to the similar lattice parameters of cubic GaAs (*a*-parameter) and tetragonal CIS and CGS (*a* and *c*/2), epitaxial growth of CIS and CGS on GaAs may occur with either a_{film} or c_{film} parameter parallel to a_{GaAs} (a_{film} // a_{GaAs} or c_{film} // a_{GaAs}, respectively). The corresponding in-plane misfit strains (*e*) are calculated as shown in Supplementary Fig. 8, assuming unit cell volume conservation: $V=(a_{bulk})^2 c_{bulk}=(a_{st})^2 c_{st}$, where *bulk* stands for bulk crystal structure and *st* stands for strained film.

The lattice parameters involved are as follows: $c_{CIS}/2 > a_{CIS} > a_{GaAs} > a_{CGS} > c_{CGS}/2$. Therefore, perfect epitaxial growth on GaAs (fully strained films) would lead to CIS under compressive strain (Supplementary Fig. 8) and CGS under tensile strain. Correspondingly, the out-of-plane d_{hkl} expands for CIS and shrinks for CGS, i.e. the diffraction of the out-of-plane reflections is shifted to lower 2θ value for CIS and higher 2θ value for CGS, compared to the respective bulk values, as shown in Supplementary Fig. 9, which also shows the expected reflection of bulk and fully strained films for the different crystallographic orientations.

Supplementary references

1. Drouin, D. *et al.* CASINO V2.42—A Fast and Easy-to-use Modeling Tool for Scanning Electron Microscopy and Microanalysis Users. *Scanning* **29,** 92–101 (2007).
2. Kaplan, L. *et al.* Synchrotron X-ray Diffraction Evidence for Native Defects in the Photovoltaic Semiconductor CuInSe2. *Adv. Mater.* **12,** 366–370 (2000).
3. Mandel, L., Tomlinson, R. D. & Hampshire, M. J. Crystal data for CuGaSe2. *Journal of Applied Crystallography* **10,** 130–131 (1977).
4. Blanton, T. N. *et al.* X-ray diffraction characterization of MOVPE ZnSe films deposited on (100) GaAs using conventional and high-resolution diffractometers. *Powder Diffraction* **24,** 78–81 (2009).
