# Supplementary material for: Sodium enhances indium-gallium interdiffusion in copper indium gallium diselenide photovoltaic absorbers
Source: Nat Commun. 2018 Feb 26;9:826. doi: 10.1038/s41467-018-03115-0 (PMC5827571; doi:10.1038/s41467-018-03115-0)
Supplement: Supplementary file 3 — Descriptions of Additional Supplementary File [file 41467_2018_3115_MOESM3_ESM.pdf]

Description of Additional Supplementary Files

File Name: Supplementary Movie 1
Description: 3D views of the atom probe tomography analyses of the CIGS films
